# Antioxidant Role and Cardiolipin Remodeling by Redox-Activated Mitochondrial Ca^2+^-Independent Phospholipase A_2_γ in the Brain

**DOI:** 10.3390/antiox11020198

**Published:** 2022-01-20

**Authors:** Pavla Průchová, Klára Gotvaldová, Katarína Smolková, Lukáš Alán, Blanka Holendová, Jan Tauber, Alexander Galkin, Petr Ježek, Martin Jabůrek

**Affiliations:** 1 Department of Mitochondrial Physiology, No. 75, Institute of Physiology of the Czech Academy of Sciences, 142 20 Prague, Czech Republic; pavla.pruchova@fgu.cas.cz (P.P.); klara.gotvaldova@fgu.cas.cz (K.G.); katarina.smolkova@fgu.cas.cz (K.S.); lukas.allan@gmail.com (L.A.); blanka.holendova@fgu.cas.cz (B.H.); jan.tauber@fgu.cas.cz (J.T.); jezek@biomed.cas.cz (P.J.); 2 Department of Biochemistry and Microbiology, Faculty of Food and Biochemical Technology, University of Chemistry and Technology, 166 28 Prague, Czech Republic; 3 Department of Biology, University of Padua, Via U. Bassi 58B, 35131 Padova, Italy; 4 Feil Family Brain and Mind Research Institute, Weill Cornell Medicine, 407 East 61st Street, New York, NY 10065, USA; alg2057@med.cornell.edu

**Keywords:** mitochondria, phospholipase iPLA_2_γ/PNPLA8, adenine nucleotide translocase, redox homeostasis, cardiolipin remodeling

## Abstract

Mitochondrial Ca^2+^-independent phospholipase A_2_γ (iPLA_2_γ/PNPLA8) was previously shown to be directly activated by H_2_O_2_ and release free fatty acids (FAs) for FA-dependent H^+^ transport mediated by the adenine nucleotide translocase (ANT) or uncoupling protein 2 (UCP2). The resulting mild mitochondrial uncoupling and consequent partial attenuation of mitochondrial superoxide production lead to an antioxidant effect. However, the antioxidant role of iPLA_2_γ in the brain is not completely understood. Here, using wild-type and iPLA_2_γ-KO mice, we demonstrate the ability of *tert*-butylhydroperoxide (TBHP) to activate iPLA_2_γ in isolated brain mitochondria, with consequent liberation of FAs and lysophospholipids. The liberated FA caused an increase in respiratory rate, which was fully inhibited by carboxyatractyloside (CATR), a specific inhibitor of ANT. Employing detailed lipidomic analysis, we also demonstrate a typical cleavage pattern for TBHP-activated iPLA_2_γ, reflecting cleavage of glycerophospholipids from both *sn*-1 and *sn*-2 positions releasing saturated FAs, monoenoic FAs, and predominant polyunsaturated FAs. The acute antioxidant role of iPLA_2_γ-released FAs is supported by monitoring both intramitochondrial superoxide and extramitochondrial H_2_O_2_ release. We also show that iPLA_2_γ-KO mice were more sensitive to stimulation by pro-inflammatory lipopolysaccharide, as reflected by the concomitant increase in protein carbonyls in the brain and pro-inflammatory IL-6 release in the serum. These data support the antioxidant and anti-inflammatory role of iPLA_2_γ in vivo. Our data also reveal a substantial decrease of several high molecular weight cardiolipin (CL) species and accumulation of low molecular weight CL species in brain mitochondria of iPLA_2_γ-KO mice. Collectively, our results support a key role of iPLA_2_γ in the remodeling of lower molecular weight immature cardiolipins with predominantly saturated acyl chains to high molecular weight mature cardiolipins with highly unsaturated PUFA acyl chains, typical for the brain.

## 1. Introduction

Cellular redox homeostasis can be defined as a dynamic steady state maintained by metabolic fluxes and redox feedback, in which oxidants (electrophiles) produced by aerobic life are reduced by antioxidant mechanisms, which reestablish nucleophilic tone [1,2]. While a spatiotemporal transient increase in oxidant production provides a means to essential regulatory redox signaling, a sustained deviation from the steady-state set point refers to physiological oxidative stress [3].

The brain is one of the main organs in the body with the highest metabolic demand and requires tight regulation of the surrounding environment [4,5]. The brain produces various reactive oxygen species (ROS) in enzymatic and non-enzymatic reactions as a byproduct of metabolism [6,7,8]. Both redox signaling and oxidative stress are recognized to be involved in all aspects of the central nervous system development, function, aging, and disease [9]. The brain deliberately produces electrophilic species to transmit redox signals in order to regulate critical functions of all its major cell types, namely glial cells (oligodendrocytes, astrocytes, microglia) and neurons [10]. Neurons are especially susceptible to harmful activities of oxidants due to their poor antioxidative equipment, and many of the reasons why the brain is vulnerable to oxidative stress remain obscure [11].

Similar to other tissues, mitochondria belong to the most important source of ROS in the brain [10,11]. There are at least eleven distinct mitochondrial sites that are known to leak electrons to oxygen to produce superoxide (O_2_^●^^−^) and downstream oxidants under different bioenergetic conditions [12]. Within the mitochondria, O_2_^●^^−^ is dismutated to H_2_O_2_ by mitochondrial matrix manganese superoxide dismutase (MnSOD) [13]. In the brain, the MnSOD activity is essential as MnSOD deficiency causes increased susceptibility to oxidative mitochondrial injury in central nervous system neurons, including severe neurodegeneration and mitochondrial oxidative damage [14,15,16]. Mitochondria also possess a multilevel network of both enzymatic and non-enzymatic antioxidant systems for the detoxification of H_2_O_2_ [17,18,19,20]. The expression and contributions of enzymatic systems for H_2_O_2_ detoxification vary widely between tissues and include catalase, glutathione/glutathione peroxidase (GSH/GPx), and the thioredoxin/peroxiredoxin systems [21]. While the expression of catalase in brain mitochondria is negligible, and the GSH/GPx shows minimal contribution [20], brain mitochondria were shown to remove H_2_O_2_ mainly in a unique respiration-dependent manner primarily via the thioredoxin 2/peroxiredoxin 3 and 5 system [20].

The role of fatty acids (FAs), notably polyunsaturated FAs (PUFAs), in the maintenance of redox homeostasis in the brain is still unclear. Similarly, the metabolism of fatty acids in the brain is still not completely understood, especially in relation to complex brain morphology [10,22,23] and the accumulation of FAs in brain tissue is associated with some inherited neurological disorders [10,11]. In addition, high unsaturated lipid content defines a cause of oxidative stress because of fatty acid-associated deleterious effects (the so-called lipotoxicity) and also the susceptibility of PUFAs to lipid peroxidation [11,24].

There are several physiological and pathological sources of elevated FA in brain tissue, including the uptake of exogenous FAs from the blood and the liberation of endogenous FAs by intracellular phospholipase A2 [10,25,26]. The phospholipase A2 (PLA2) superfamily contains more than 50 enzymes in mammals that are subdivided into several distinct families on a structural and biochemical basis, including group VI, also termed patatin-like phospholipase domain-containing lipases (PNPLAs) [26]. The phospholipases iPLA_2_β (group VIA PLA2, PNPLA9) and iPLA_2_γ (group VIB, PNPLA8) were both shown to be located to mitochondria; however, only iPLA_2_γ is known to contain the N-terminal mitochondrial localization sequence [26,27]. Like all PNPLAs, the mitochondrial iPLA_2_γ catalyzes the cleavage of acyl groups from glycerophospholipids from both *sn*-1 and *sn*-2 positions and is predominantly an *sn*-1 lipase for phospholipids containing a PUFA chain at the *sn*-2 position [28]. iPLA_2_γ is also a major mediator releasing oxidized aliphatic chains from cardiolipin, and iPLA_2_γ together with cardiolipin play integrated roles in lipid second-messenger production and mitochondrial bioenergetics during oxidative stress [29]. In addition, studies utilizing iPLA_2_γ-knockout (KO) mice identified the obligatory role of iPLA_2_γ in neuronal mitochondrial structure and function [30]. iPLA_2_γ-KO mice are characterized by significant changes in hippocampal lipids, including cardiolipin content and molecular species composition, and the presence of increased levels of oxidized lipid molecular species [30]. In addition, decreasing iPLA_2_γ activity increases lipid oxidative stress accompanied by mitochondrial disorders in the brain [31]. Thus, neurodegeneration is strongly related to iPLA_2_γ activity [32], and upregulating iPLA_2_γ activity can restore mitochondrial membrane and function [31,33].

Our previous results revealed a non-canonical role for iPLA_2_γ in participating in cellular antioxidant protection. We showed that the iPLA_2_γ is directly stimulated by hydroperoxides and hence can be activated physiologically by redox signaling or oxidative stress [34,35,36]. The *tert*-butylhydroperoxide (TBHP) or H_2_O_2_-activated iPLA_2_γ liberates FAs, which interact with mitochondrial uncoupling proteins (UCPs) and adenine nucleotide translocases (ANTs). This induces FA-mediated H^+^ transport, leading to a mild dissipation of the mitochondrial protonmotive force (also termed mild mitochondrial uncoupling) [37,38,39,40,41,42,43,44]. This leads to the attenuation of mitochondrial superoxide formation ([34,35,36], reviewed in [45,46]). Therefore, iPLA_2_γ is a crucial component of a feedback regulation, where partial ROS increase is subsequently eliminated by mitochondrial uncoupling due to activation of the iPLA_2_γ-UCP(ANT) antioxidant synergy [45,46].

Here, we tested the hypothesis that mitochondrial iPLA_2_γ participates in the maintenance of redox homeostasis in the brain due to the interaction of iPLA_2_γ-released FAs with brain UCPs or ANT. Using wild-type (WT) and iPLA_2_γ-KO mice, we demonstrate the ability of TBHP to activate iPLA_2_γ in isolated brain mitochondria, with consequent liberation of FAs and lysophospholipids. The liberated FA caused an increase in respiratory rate, which was fully inhibited by carboxyatractyloside (CATR), a specific inhibitor of ANT. Employing detailed lipidomic analysis, we also demonstrate a typical cleavage pattern for TBHP-activated iPLA_2_γ, reflecting cleavage of glycerophospholipids from both *sn*-1 and *sn*-2 positions releasing free saturated FAs, monoenoic FAs, and predominant PUFAs. The acute antioxidant role of iPLA_2_γ is supported by monitoring both intramitochondrial superoxide and extramitochondrial H_2_O_2_ release and is further supported by the measurement of protein carbonyl content in brain tissue and IL-6 concentration in the serum. Moreover, iPLA_2_γ participation in remodeling of cardiolipins was suggested by the predominance of lower-molecular-weight cardiolipins containing relatively saturated acyl chains in mitochondria of iPLA_2_γ-KO mice, reflecting stalled deacylation of immature cardiolipins and their subsequent reacylation/transacylation into mature cardiolipins containing long PUFA acyl chains.

## 2. Materials and Methods

### 2.1. Chemicals and Reagents

r-Bromenol lactone (r-BEL) and carboxyatractyloside (CATR) were obtained from Cayman Chemical (Ann Arbor, MI, USA). Amplex Red and MitoSOX Red^TM^ were purchased from Thermo Fisher Scientific (Waltham, MA, USA). Other reagents were obtained from Sigma-Aldrich (Darmstadt, Germany).

### 2.2. Creation of iPLA_2_γ/PNPLA8 Knockout Mice

The iPLA_2_γ/PNPLA8 knockout mice were generated using transcription activator-like effector nucleases (TALENs) as described previously [47], starting with C57Bl6/N mice. TALENs were designed to target the *Pnpla8* exon-3 to eliminate the XbaI restriction site (Supplemental Figure S1). DNA from tails of young mice was analyzed by the PCR restriction-fragment-length polymorphism. Purified PCR products were digested with XbaI (ThermoFisher, Waltham, MA, USA). To further select appropriate mice for breeding, the PCR products were subcloned into the pGEM^®^-T Easy Vector System (Promega, Madison, WI, USA) and sequenced using M13 reverse and forward sequencing primers. For further breeding, mice bearing a 13 base-pairs long deletion in exon-3, which resulted in the formation of a premature stop codon in the fourth exon, were selected. The wild-type (WT) mice used were those backcrossed >10 generations into the iPLA_2_γ/PNPLA8-knockout mice background. Animals were housed in open cages and kept at standard housing conditions with free access to water and a normal chow diet (NCD; Altromin 1314 Forti, Postfach, Germany). All animal studies were ethically reviewed and performed in accordance with European Directive 2010/63/EU, complying with the NIH Publication No.85–23 (revised 1996) and the ARRIVE guidelines; and were approved by the Czech Central Commission for Animal Welfare.

### 2.3. Isolation of Mitochondria

Typically, 2–4 mice 20–24 weeks old were sacrificed by cervical dislocation. The brains were removed and washed in ice-cold isolation medium (225 mM of mannitol, 75 mM of sucrose, 5 mM of HEPES, 1 mM of EGTA, 0.1 mM of EDTA, pH 7.4 by TRIS) supplemented with 0.5% bovine serum albumin (BSA) and with 5 mM of N-acetylcysteine (NAC), where indicated. Brain mitochondria were isolated following the procedure of Stepanova et al. [48], combining differential centrifugation and digitonin treatment. Briefly, after homogenization with a Dounce homogenizer, homogenates were centrifuged at 2000× *g* for 10 min at 4 °C. Pellets were discarded, and 0.02% digitonin was added to supernatants for synaptosome lysing. Supernatants were centrifuged at 10,000× *g* for 10 min at 4 °C, pellets were resuspended in the isolation medium lacking BSA and NAC, and were centrifuged as above. The final pellet was resuspended to an approximate concentration of 20 mg protein/mL in the isolation medium lacking BSA. The exact protein concentration was determined by the BCA method (Sigma).

### 2.4. High-Resolution Respirometry

Mitochondrial oxygen consumption was monitored using an Oxygraph 2k high-resolution respirometer (Oroboros, Innsbruck, Austria) after air calibration and background correction. The respiratory medium included KCl (125 mM), HEPES (20 mM), EGTA (0.2 mM), KH_2_PO_4_ (2 mM), MgCl_2_ (2 mM), pH 7.4 at 30 °C. The respiratory medium routinely contained both Complex I- and Complex II-linked substrates, glutamate (5 mM), malate (1 mM), and succinate (5 mM), referred to as GMS, and ATP synthase inhibitor oligomycin (1 μg/mL), where applicable. A low non-saturating concentration of BSA (typically titrated to 0.5 μM) was also used during the experiments to minimize the effect of residual contaminating FAs.

### 2.5. Intramitochondrial Detection of Superoxide Formation

Surplus superoxide released into the mitochondrial matrix was monitored by increasing fluorescence of MitoSOX Red^TM^ (Thermo Fisher), which accumulates in the mitochondrial matrix [35,36,49]. To support a uniform distribution of MitoSOX Red within mitochondria, the isolated brain mitochondria at a concentration of 0.2 mg/mL were preincubated with 1 μM of MitoSOX and 5 mM of NAC in respiration buffer containing respiratory substrates (only 5 mM of glutamate, 1 mM of malate) for 45 min. After this preincubation, 0.5% BSA was added for another 5 min to remove any residual released FAs. Mitochondria were centrifuged at 10,000× *g* for 10 min at 4 °C, and pellets were resuspended in the isolation medium. Fluorescence was monitored with an RF 5301 PC spectrofluorometer (Shimadzu, Duisburg, Germany) at excitation of 510 nm and emission of 590 nm at 30 °C.

### 2.6. Extramitochondrial Detection of H_2_O_2_ Release

The H_2_O_2_ released out of mitochondria was detected with Amplex^TM^ UltraRed Reagent (ThermoFisher), which allows for a highly sensitive and selective quantitative enzymatic detection of H_2_O_2_ [50]. Mitochondria were used at a concentration of 0.2 mg/mL protein in a standard respiration medium at 30 °C, and the monitoring of H_2_O_2_ release was initiated by adding the Amplex UltraRed probe (10 μM) in the presence of horseradish peroxidase (5 U/mL). The fluorescence signal was calibrated for each experimental condition by sequential additions of H_2_O_2_ aliquots. Fluorescence intensity was detected using excitation and emission wavelengths of 572 nm and 580 nm on an RF 5301 PC spectrofluorometer (Shimadzu) at 30 °C.

### 2.7. LC–MS-Based Lipidomic Profiling

Extraction was carried out using a biphasic solvent system of cold methanol, methyl *tert*-butyl ether (MTBE), and water. In more detail, 0.4 mg of the mitochondrial pellet was homogenized (1.5 min) with 275 µL of methanol and 275 µL of 10% methanol using a grinder. Then, 1 mL of MTBE was added, and the tubes were shaken (1 min) and centrifuged (16,000 rpm, 5 min, 4 °C). For lipidomic profiling, 500 µL of upper organic phase was collected, evaporated, and resuspended using methanol containing 12-[[(cyclohexylamino) carbonyl]amino]-dodecanoic acid (CUDA) internal standard, shaken (30 s), centrifuged (16,000 rpm, 5 min, 4 °C), and used for LC-MS analysis in negative electrospray ion mode.

The LC-MS analysis systems consisted of a Vanquish UHPLC System (Thermo Fisher) coupled to a QExactive Plus mass spectrometer (Thermo Fisher). Experimental conditions are described in detail in [51]. A sample volume of 5 μL was used for injection. LC-MS instrumental files from lipidomic profiling were processed through MS-DIAL 4.70 software [52], including lipid annotations. Exported raw data were filtered using blank samples, serial dilution samples, and quality control (QC) pool samples with relative standard deviation (RSD) < 30%, normalized using the LOESS approach by means of QC pool samples, regularly injected between 10 actual samples. Samples were randomized across the platform run. Only lipid species with a signal intensity 10-fold higher than in corresponding blank samples were considered for further data analysis.

### 2.8. Agent/Drug Application to Mice

Where indicated, mice were injected with lipopolysaccharides (LPS) (6 mg/kg body weight) or with iPLA_2_γ inhibitor r-BEL (1 mg/kg body weight) and LPS in parallel. LPS was administered one time intraperitoneally (i.p.) 6 h before sacrifice, while r-BEL was subcutaneously applied to mice in 24-h periods, three times during 72 h [33]. Animals treated with vehicle (phosphate-buffered saline and/or DMSO) under the same conditions were used as the control group. Mice were sacrificed, selected tissues were collected, and aliquots of each tissue were immediately frozen in liquid nitrogen and stored at −80 °C until the analysis was performed. Interleukin-6 (IL-6) concentrations were determined in mouse sera. The protein carbonyl content was established from tissue homogenates. Typically, one group contained 3–4 mice, gender-mixed.

### 2.9. Tissue Homogenates

The selected tissues were minced into small pieces in the mitochondrial isolation medium and homogenized using a Potter-Elvegen tissue grinder. The homogenates were centrifuged at 10,000× *g* for 10 min at 4 °C, and the supernatants were stored in aliquots at −80 °C. For protein carbonylation, the supernatant absorbance at 280 and 260 nm was checked to determine the possible presence of nucleic acids in the sample. As the ratio of 280/260 nm was less than 1, samples were incubated with streptomycin sulfate at a final concentration of 1% in the sample [53]. After 15 min of incubation at room temperature, the samples were centrifuged at 6000× *g* for 10 min at 4 °C, and the supernatants were used for determining protein carbonyl content.

### 2.10. Quantification of Interleukin-6 Levels

IL-6 levels were determined from collected sera using a commercially available kit (IL-6 Mouse ProQuantum Immunoassay Kit, Invitrogen, Waltham, MA, USA). The high-sensitivity immunoassay utilizes proximity ligation assay technology to combine antibody-antigen binding specificity and amplification capabilities of real-time PCR. Briefly, mouse sera were incubated with antibody conjugated to a DNA oligonucleotide for 1 h at room temperature according to manufacturer instructions. After incubation, ligase was added to the samples for the creation of a template strand for amplification. A qPCR reaction was performed on the Bio-Rad CFX96 instrument (Bio-Rad, Hercules, CA, USA).

### 2.11. Quantification of Protein Carbonyls

The samples were prepared using the method developed by Levine et al. [53] with modifications. Briefly, samples with protein concentrations in the range of 1–10 mg/mL of tissue homogenates were treated with 0.8 mL of 20 mM 2,4-dinitrophenylhydrazine (DNPH) in 2.5 M of HCl for 60 min at room temperature in the dark. The reaction was stopped by adding 1 mL of 20% trichloroacetic acid (TCA), and the TCA-treated samples were incubated on ice for 5 min and centrifuged at 10,000× *g* for 10 min at 4 °C. The pellets were resuspended in 1 mL of 10% TCA, incubated on ice for 5 min, and then centrifuged as above. The precipitates were washed three times in 1 mL of 1:1 ethanol: ethyl acetate solution and centrifuged as above. The final pellet was dissolved in 0.5 mL of 6 M guanidine hydrochloride. The total protein carbonyl content was measured at 25 °C following the absorbance at 370 nm and determined using the molar absorption coefficient of 22,000 M^−1^ cm^−1^.

### 2.12. Statistical Analysis

The lipidomic data were normalized to total-ion current (TIC) before subsequent analysis. The multivariate analyses were performed using MetaboAnalyst 5.0 [54]. Hierarchical clustering analysis (Euclidean distance) was performed using ComplexHeatmap (Bioconductor) package by RStudio version 1.4. [55] with log10-transformed data scaled with Pareto scaling [56]. The remaining statistical analysis was performed using the GraphPad Prism software version 5.01. The difference between two groups was analyzed using the unpaired Student’s *T*-test, while two-way analysis of variance (ANOVA) followed by Bonferroni’s post-test was used for comparison of multiple groups. Differences with *p* < 0.05 were considered statistically significant.

## 3. Results

### 3.1. Fatty Acid-Induced Increase in Respiration following Redox Activation of iPLA_2_γ

#### 3.1.1. Uncovering the Redox Activation of iPLA_2_γ in Isolated Brain Mitochondria

**Figure 1:**
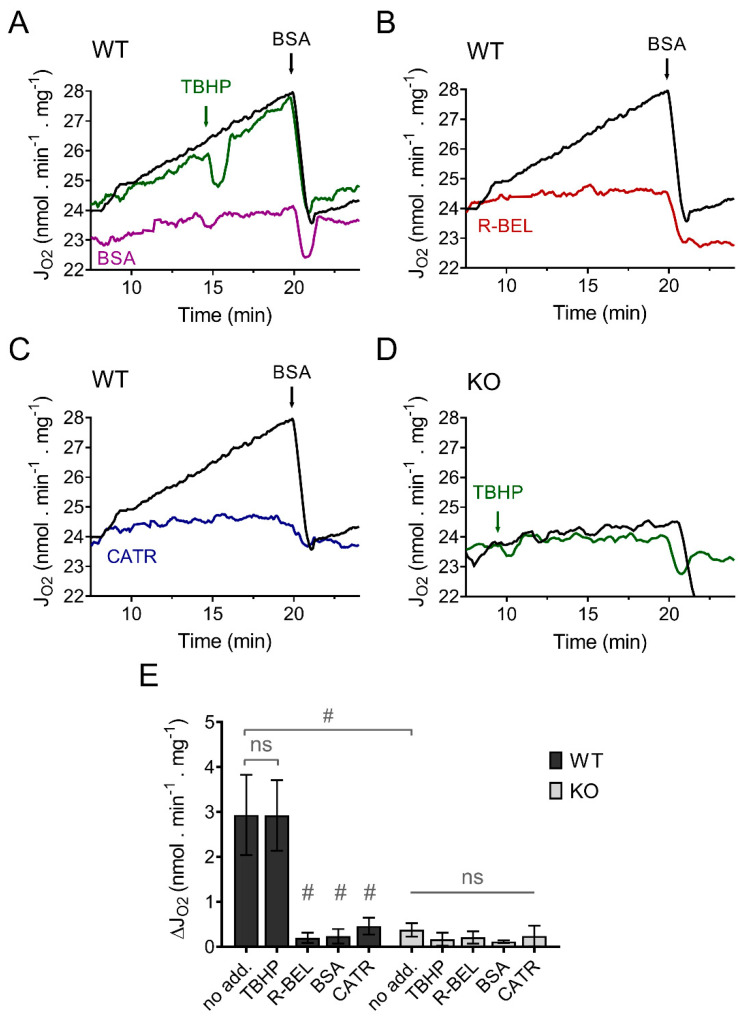
iPLA_2_γ is intrinsically active in brain mitochondria isolated from wild-type mice. Representative traces are shown for state-4 respiration rates (J_O2_) evolving in time in the presence of 5 mM of glutamate, 1 mM of malate, and 5 mM of succinate (GMS). (**A**) Spontaneous increase in the rate of respiration (black trace) was not sensitive to TBHP (green trace) but was prevented by BSA (purple trace). (**B**) The effect of r-BEL (red trace). (**C**) Effect of CATR (blue trace). (**D**) The basal state-4 respiration rate (black trace) and the effect of TBHP (green trace) in mitochondria isolated from iPLA_2_γ-KO mice. (**E**) The effects of various reagents on the respiration rates of brain mitochondria isolated from WT (black bars) or iPLA_2_γ-KO mice (grey bars) monitored by changes (Δ) in O_2_ flux (J_O2_). Reagent concentrations: TBHP, 50 µM; BSA, 5 µM; r-BEL, 2 µM; CATR, 2 µM. All inhibitors were added before the addition of TBHP. Values are plotted as means ± standard deviations. The number of replicates and isolations was at least 4. # *p* < 0.001 compared to no addition (no add). ns, No significant differences were found between the rates in mitochondria isolated from iPLA_2_γ-KO mice.

In contrast to our previous results using mitochondria isolated from the heart, lung, and spleen, which revealed a TBHP-dependent increase in respiration due to activation of iPLA_2_γ [34,36], isolated murine brain mitochondria displayed a steady increase in the rate of basal respiration without the obligatory requirement for TBHP (Figure 1A). This increase in respiratory rate was entirely prevented by an excess of bovine serum albumin (Figure 1A), by a selective iPLA_2_γ inhibitor r-BEL (Figure 1B), and also by CATR (Figure 1C). On the contrary, no such increase in respiration was observed in brain mitochondria isolated from iPLA_2_γ-KO mice (Figure 1D). These results indicate that the observed increase in the respiratory rate can be attributed to FAs being gradually liberated by iPLA_2_γ during the experiment and that iPLA_2_γ in isolated brain mitochondria is intrinsically active. The CATR inhibition also shows that the liberated FAs interact with ANT to increase the H^+^ conductance and, consequently, the respiratory rate.

An additional detailed analysis of the coupling states and respiration rates of the mitochondria isolated from WT and iPLA_2_γ-KO mice, performed in the presence of BSA in excess (0.1%), revealed that both behave identically under the given experimental conditions (Supplemental Figure S2). Specifically, both mitochondrial isolations have nearly identical rates of basal respiration following the addition of substrates, have identical state-3 respiration determined by the addition of ADP (OXPHOS capacity), and have similar maximal respiratory rates obtained by titration of the uncoupler FCCP (maximal respiratory chain capacity) (Supplemental Figure S2). Moreover, the addition of 0.5 μM of linoleic acid to the respiring isolated brain mitochondria also led to an identical increase in respiration in both preparations from WT and iPLA_2_γ-KO mice, and this increase was abolished by BSA and CATR (Supplemental Figure S3). Altogether, these results show that brain mitochondria isolated from WT and iPLA_2_γ-KO mice are similar in respect to their respiratory properties and the ability of FAs to interact with ANT and increase H^+^ conductance.

**Figure 2:**
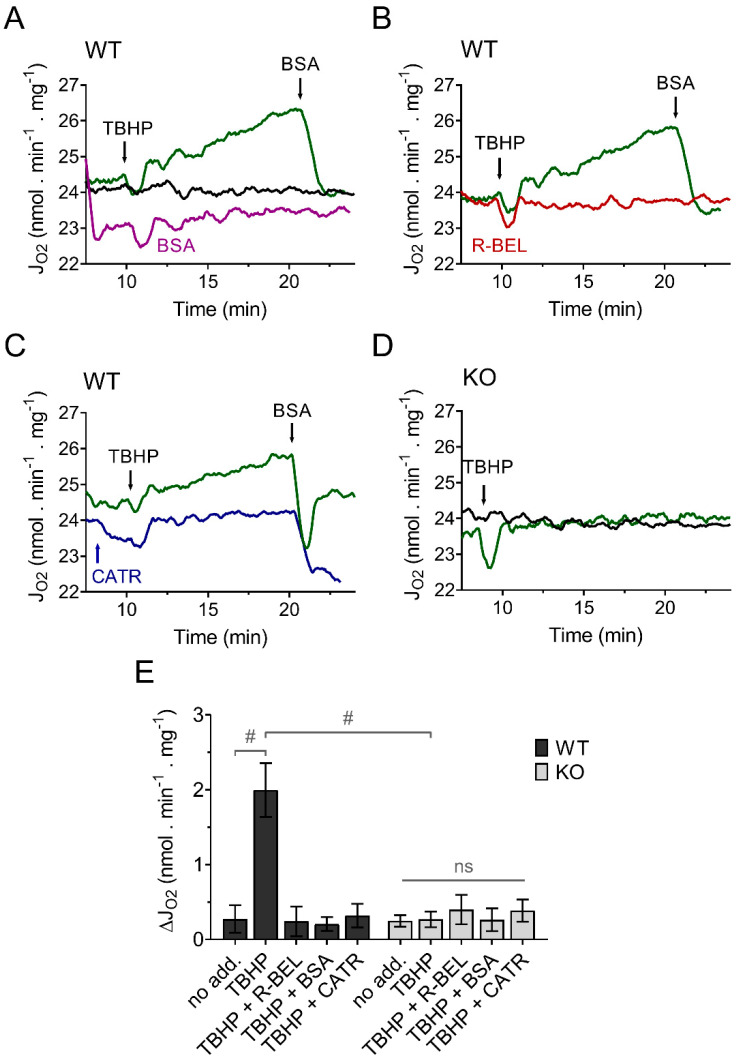
TBHP activates iPLA_2_γ in brain mitochondria isolated in the presence of N-acetyl cysteine. Representative traces are shown for state-4 respiration rates (J_O2_) evolving in time in the presence of 5 mM glutamate, 1 mM malate, and 5 mM succinate. (**A**) The basal respiration rate (black trace) was increased by TBHP (green trace), and the TBHP-induced increase in the respiration rate was prevented by BSA (purple trace). (**B**) The effect of r-BEL (red trace). (**C**) Effect of CATR (blue trace). (**D**) The basal state-4 respiration rate (black trace) and the effect of TBHP (green trace) in mitochondria isolated from iPLA_2_γ-KO mice. (**E**) The effects of various reagents on the respiration rate of brain mitochondria isolated from WT (black bars) or iPLA_2_γ-KO mice (grey bars) monitored by changes (Δ) in O2 flux (J_O2_). Reagent concentrations were identical to those described in Figure 1. The number of replicates and isolations was at least 4 for both WT and iPLA_2_γ-KO. # *p* < 0.001 compared to no addition (no add). ns, No significant differences between the rates were found in mitochondria isolated from iPLA_2_γ-KO mice.

Because we previously demonstrated that the recombinant reconstituted iPLA_2_γ is sensitive to thiol-reducing compounds [35], we expected that the presence of a membrane-permeable thiol reducing compound, such as N-acetyl cysteine (NAC), during mitochondrial isolation, could reduce the intrinsically active brain mitochondrial iPLA_2_γ and thus restore the ability of iPLA_2_γ to respond to and be activated by external or internal oxidants. The results shown in Figure 2. support our prediction. Brain mitochondria isolated from WT mice in the presence of NAC show a negligible increase in the basal respiratory rate, but the addition of TBHP led to a steady increase in the rate of basal respiration (Figure 2A), comparable to the increase seen in the absence of NAC. Analogously, this increase in respiratory rate was completely prevented by a BSA excess (Figure 2A), by a selective iPLA_2_γ inhibitor r-BEL (Figure 2B), and also by CATR (Figure 2C). Again, no such increase in respiration was observed in brain mitochondria isolated from iPLA_2_γ-KO mice (Figure 2D). These results are consistent with the ability of iPLA_2_γ to be reversibly inhibited by thiol reducing compounds, either directly or indirectly. They support the hypothesis of redox activation of iPLA_2_γ by oxidative posttranslational modification of accessible protein thiols [46].

**Figure 3:**
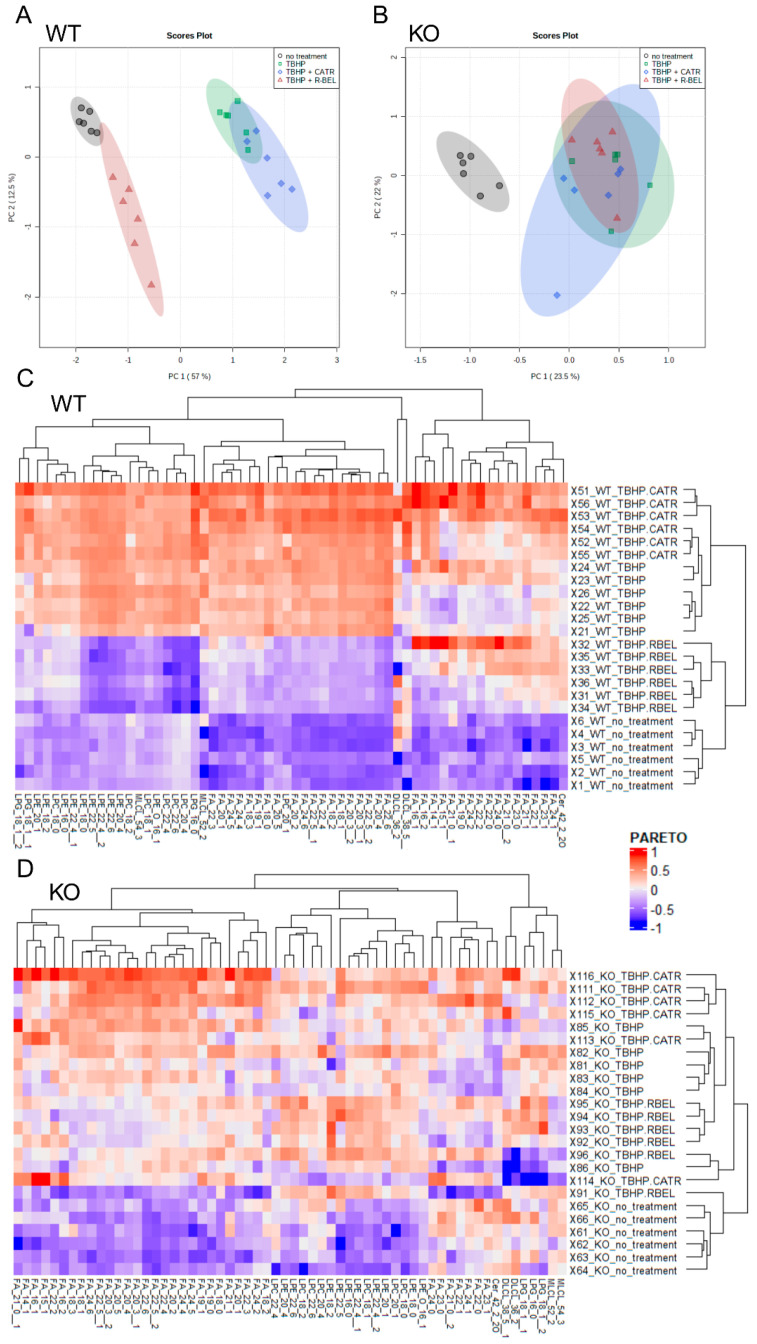
Lipidomic analysis of isolated WT and iPLA_2_γ-KO mouse mitochondria. Principal component analysis scores plots (PCA) of TIC-normalized log10-transformed intensities for WT (**A**), iPLA_2_γ-KO (**B**). (**C**,**D**) Heatmap based on hierarchical clustering presented in dendrograms according to the Euclidean distance. Data are log10-transformed and scaled by Pareto scaling. Lipids with loadings values in PC1 > 0.05 in WT (**C**) and iPLA_2_γ-KO (**D**) mouse brain mitochondria. Isomers are marked with the number after double underscores. Lipidomic data were collected from *n* = 6 replicates.

Next, to obtain a complete reaction pattern of TBHP-stimulated catalytic activities of iPLA_2_γ, mitochondria isolated in the presence of NAC were allowed to respire for 1 h under the conditions described in Figure 2, and the subsequently isolated mitochondrial pellets were subjected to unbiased LC-MS lipidomic profiling. First, to reveal patterns in the data, we performed a multivariate principal component analysis (PCA) based on amounts of lipid species normalized to total ion current (TIC) for all experimental conditions (Figure 3A,B). The score plots show the similarity of phenotypically similar groups and the variability of remote groups, and in this case, active vs. inactive iPLA_2_γ. In WT brain mitochondria, the result of this analysis formed two related clusters. As shown in Figure 3A,C, the group of “TBHP + r-BEL” clustered together with the “no treatment” group (mitochondria frozen immediately following the isolation and not subjected to the TBHP treatment), which indicates that both groups demonstrate a common metabolic phenotype, where iPLA_2_γ is not activated. On the contrary, the groups of “TBHP” and “TBHP + CATR” formed another cluster, which indicates that these two groups represent metabolic phenotype following the iPLA_2_γ activation. Simply, they represent cleaved products of iPLA_2_γ, liberated FAs, and lysophospholipids. Furthermore, no such clustering was found in iPLA_2_γ-KO brain mitochondria (Figure 3B,D). The PCA analysis also revealed a separation of WT and iPLA_2_γ-KO samples in PC1 (Supplemental Figure S4), which indicates that there exists a long-term effect of the iPLA_2_γ ablation on the overall lipid profile besides the changes initiated by the respective acute treatments (see Section 3.2 below).

#### 3.1.2. Detailed Lipidomic Analyses Reveal the Main Products of the TBHP-Activated iPLA_2_γ in Brain Mitochondria

Subsequently, we analyzed metabolites, which were responsible for sample clustering within PC1 (Supplemental Figure S5). Hierarchical clustering analysis of metabolites with loading values in PC1 > 0.05 is presented in Figure 3C,D. Importantly, upregulated metabolites in the treatment groups in WT samples distinguished clusters of free FAs and several groups of lysophospholipids, which were absent following the r-BEL treatment (Figure 3C). This type of clustering was completely missing in iPLA_2_γ-KO samples (Figure 3D). Therefore, the WT pattern “TBHP” and “TBHP + CATR” should indicate the iPLA_2_γ-specific products.

Moreover, several saturated FAs were liberated after the TBHP treatment, such as 18:0, 19:0, 23:0, 24:0, 22:0, and 21:0 (ordered by descending PC1 loadings values) (Supplemental Figure S4). These data independently support the conclusion that the phospholipase cleaving these FAs by its PLA1 activity is a member of the PNPLA family of group VI because this group possesses the ability to cleave *sn*-1 positions of the glycerol backbone occupied typically with saturated FAs. We conclude that the identified species of lysophosphatidylcholines (LPCs), lysophosphatidylethanolamines (LPEs), lysophosphatidylglycerols (LPGs), monolysocardiolipins (MLCLs), and dilysocardiolipins (DLCLs) are explicitly formed by the TBHP-induced activity of iPLA_2_γ in brain mitochondria.

#### 3.1.3. Univariate Analyses of Lipidomic Data Demonstrate a More Detailed Pattern of iPLA_2_γ Reaction Products

**Figure 4:**
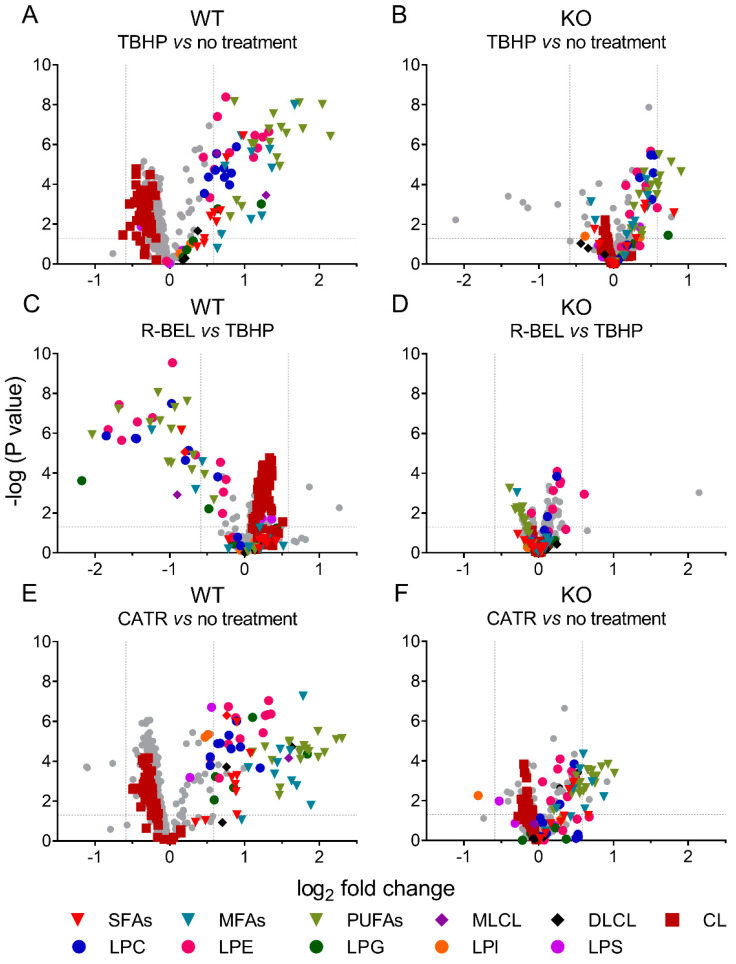
Volcano plot analysis of TBHP-induced, iPLA_2_γ-dependent cleavage of phospholipids. Brain mitochondria isolated from WT mice and respiring in the presence of TBHP are related to mitochondria with no treatment frozen immediately after isolation (**A**) and to mitochondria treated with TBHP + r-BEL (**C**). Analogously, brain mitochondria isolated from iPLA_2_γ-KO mice and respiring in the presence of TBHP are related to mitochondria with no treatment frozen immediately after isolation (**B**) and to mitochondria treated with TBHP + r-BEL (**D**). TBHP + CATR treatment is related to mitochondria with no treatment frozen immediately after isolation in WT (**E**) and iPLA_2_γ-KO (**F**) mice. Abbreviations: SFAs, saturated FAs; MFAs, monoenoic FAs; PUFA, polyunsaturated FAs; MLCL, monolysocardiolipin; DLCL, dilysocardiolipin; CL, cardiolipin; LPC, lysophosphatidylcholine; LPE, lysophosphatidylethanolamine; LPG, lysophosphatidylglycerol; LPI, lysophosphatidylinositol; LPS, lysophosphatidylserine. Lipidomics data were collected from *n* = 6 replicates.

Either considering multivariate analyses with PC1 loadings > 0.05 (Supplemental Figure S4) or comparing two selected groups using univariate analyses, we further examined the differences between mitochondria isolated from WT and iPLA_2_γ-KO mice in more detail (Figure 4). In agreement with the multivariate analyses, the addition of TBHP significantly increased the relative concentration of free saturated, monoenoic, and polyunsaturated FAs, MLCL, and lysophospholipids (LPC, LPG, LPE), while it decreased the relative levels of cardiolipins (CL) (Figure 4A). The addition of r-BEL inhibited the TBHP-dependent iPLA_2_γ activation in WT mitochondria (Figure 4C), resulting in a similar pattern to that obtained with iPLA_2_γ-KO mice (Figure 4B,D). Surprisingly, more products, including DLCL and lysophospholipids LPS and LPI, were detected when CATR was used together with TBHP in WT brain mitochondria (Figure 4E). This pattern ceased for CATR plus TBHP treatment of iPLA_2_γ-KO samples (Figure 4F).

**Figure 5:**
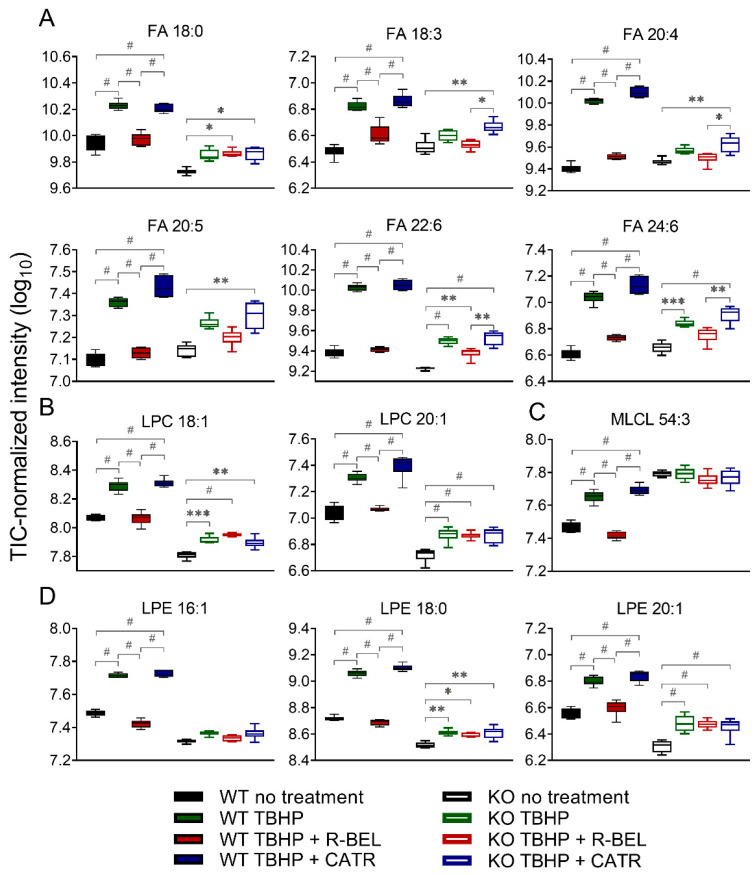
The changes in selected fatty acids and lysophospholipids of isolated WT and iPLA_2_γ-KO mouse mitochondria. (**A**) Fatty acids, (**B**) lysophosphatidylcholines, (**C**) monolysocardiolipin, (**D**) lysophosphatidylethanolamines. * *p* < 0.05; ** *p* < 0.01; *** *p* < 0.005; # *p* < 0.001. Lipidomic data are collected from *n* = 6.

Next, we focused on those lipid species in the WT group, which were consistently downregulated in the presence of r-BEL and were not affected by r-BEL treatment in the iPLA_2_γ-KO group. In Figure 5, we present 12 of the most prominent lipid metabolites that fulfilled such requirements and were positively correlated with the TBHP-induced iPLA_2_γ activity in brain mitochondria. These include stearic acid (C18:0), α-linolenic acid (18:3), arachidonic acid (C20:4), eicosapentaenoic acid (C20:5), docosahexaenoic acid (C22:6), and tetracosahexaenoic acid (C24:6) (Figure 5A). Specifically, the addition of TBHP resulted in an approximate 4.8-fold increase in C22:6 (*p* < 0.0001, *n* = 6), a 4.5-fold increase in C20:4, while C24:6, C22:4, C20:5, and C18:0 were increased more than 3-fold. Again, the presence of cleaved stearic acid, an unsaturated FA, is consistent with the unique ability of PNPLAs, i.e., group VI phospholipases, to cleave the *sn*-1 side chain of phospholipids. The overall pattern of FAs released from both *sn*-1 (predominantly SFAs) plus *sn*-2 cleaved positions (predominantly PUFAs) is consistent with the lipid signatures typical to brain mitochondria [57].

The corresponding parallel increase in certain lysophospholipids species was also found, namely lysophosphatidylcholines (LPC 18:1, LPC 20:1, Figure 5B), monolysocardiolipin (MLCL 54:3, Figure 5C), and lysophosphatidylethanolamines (LPE 16:1, LPE 18:0, and LPE 20:1, Figure 5D), which, again, reflect the pattern of iPLA_2_γ preferential activity. In brain mitochondria isolated from iPLA_2_γ-KO mice, the changes in the selected FAs and lysophospholipids were much less prominent, or they were absent altogether (Figure 5 A–D). These residual TBHP-induced changes observed in iPLA_2_γ-KO may reflect activities of other mitochondria-located phospholipases, such as iPLA_2_β, or may also possibly reflect the presence of contaminant cytosolic phospholipases.

### 3.2. Differences in Brain Mitochondrial Lipid Composition of iPLA_2_γ- KO Mice

#### Mitochondrial iPLA_2_γ Participates in Cardiolipin Remodeling

**Figure 6:**
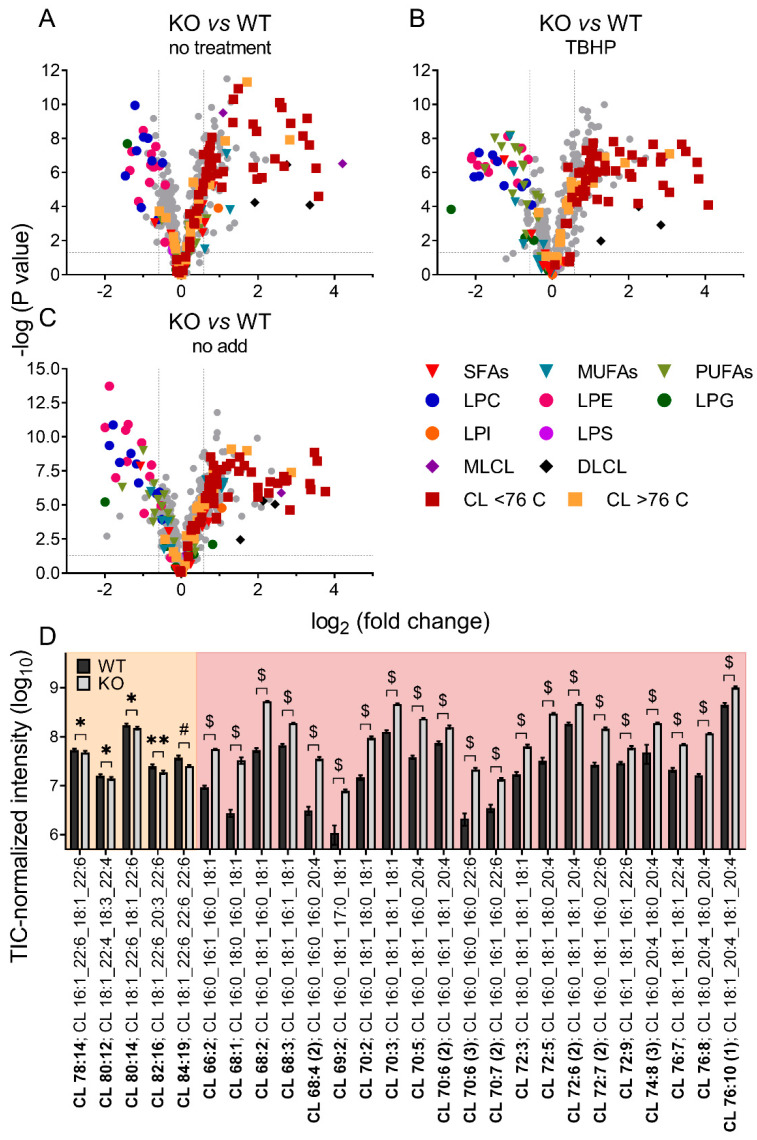
Differences in cardiolipin composition for brain mitochondria from iPLA_2_γ-KO mice vs. WT. (**A**) Isolated mitochondria without further treatment, frozen instantly after isolation, (**B**) mitochondria isolated in the presence of NAC and respiring for 1 h in the presence of TBHP, and (**C**) mitochondria isolated in the absence of NAC and respiring for 1 h in the absence of TBHP. (D) The changes in cardiolipin composition of individual cardiolipin species of isolated WT and iPLA_2_γ-KO mitochondria, without further treatment, frozen instantly after isolation (**D**). Isomers are marked with the number in parentheses. * *p* < 0.05; ** *p* < 0.01; # *p* < 0.001; $ *p* < 0.000001. Lipidomic data are collected from *n* = 6 replicates.

Unbiased lipidomics also revealed profound differences in the cardiolipin species profile for mitochondria of WT and iPLA_2_γ-KO mice. Figure 6 shows a detailed analysis of CL species where the iPLA_2_γ-KO mouse brain mitochondria were related to WT under several experimental conditions. First, we analyzed the CL species profile of isolated brain mitochondria that were not subjected to any further treatment and were frozen immediately after the isolation (Figure 6A). We further analyzed the CL species profile following the respiration of NAC-treated mitochondria and inducing the iPLA_2_γ activity by TBHP (Figure 6B), and following the respiration of mitochondria without the NAC treatment, containing the intrinsically active iPLA_2_γ (Figure 6C). In all tested conditions, numerous specific species of C66 to C76 cardiolipins were elevated in mitochondria isolated from iPLA_2_γ-KO mice. Detailed compositions of CL acyl chains of isolated iPLA_2_γ-KO brain mitochondria that were not subjected to any further treatment show a substantial decrease of five high molecular weight CL species (CL78:14, CL80:12, CL80:14, CL82:16, and CL84:19) and, in contrast, several-fold accumulation of many species of lower molecular weight CLs (Figure 6D). The existence of these elevated cardiolipin species in iPLA_2_γ-KO relative to WT mouse brain mitochondria could be explained by a stalled specific deacylation of CL acyl chains due to the iPLA_2_γ ablation. Since CL68:1 is likely to be synthesized from phosphatidylglycerol PG {18:0,18:1} and 1,2-dihexadecanoyl-*sn*-glycero-3-cytidine-5′-diphosphate (CDP-DG {16:0,16:0}), or from PG {16:0,18:1} and CDP-DG {18:0,16:0}, without activity of iPLA_2_γ, cardiolipin is no longer deacylated in its C18:0 and C16:0 acyl chains and remains unmodified in the brain tissues of iPLA_2_γ-KO mice.

These data suggest that phospholipase iPLA_2_γ participates in the deacylation of lower molecular weight immature cardiolipins with predominantly saturated acyl chains to high molecular weight mature cardiolipins with highly unsaturated PUFA acyl chains, typical for the brain.

### 3.3. Antioxidant Role of iPLA_2_γ-Released Fatty Acids in Isolated Brain Mitochondria

#### 3.3.1. Mitochondrial Superoxide Release into the Matrix Decreases following Redox Activation of iPLA_2_γ

**Figure 7:**
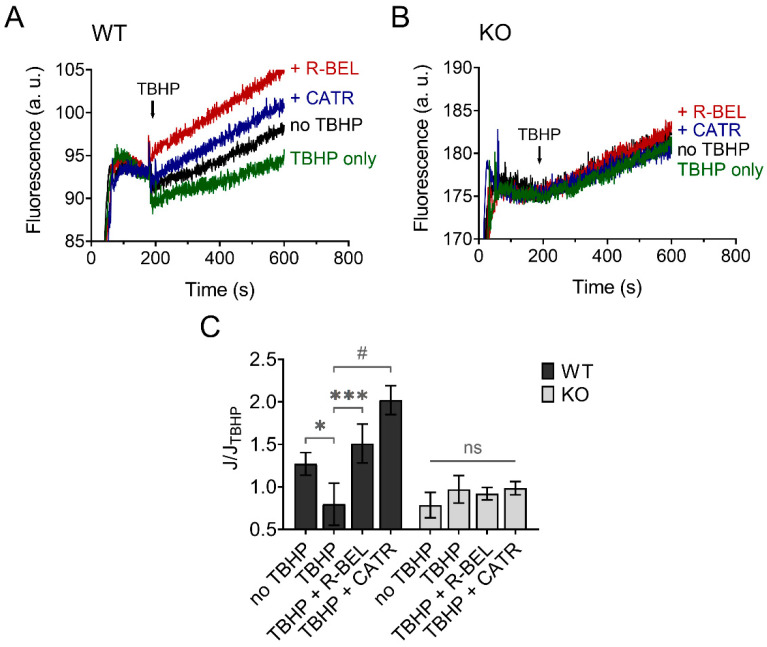
TBHP-dependent activation of iPLA_2_γ leads to decreased superoxide formation. Representative traces of MitoSOX fluorescence in isolated brain mitochondria respiring in the presence of 5 mM of glutamate, 1 mM of malate, and 5 mM of succinate. (**A**) The basal rate of superoxide release (black trace) was decreased by TBHP (green trace), and the TBHP-induced effect was reversed by r-BEL (red trace) and CATR (blue trace). (**B**) The basal rate of superoxide release (black trace), the effect of TBHP (green trace), r-BEL (red trace), and CATR (blue trace) in mitochondria isolated from iPLA_2_γ-KO mice. (**C**) The effects of tested compounds on the rates of superoxide release (J) relative to the rate observed in the presence of TBHP (J_TBHP_) of brain mitochondria isolated from WT (black bars) or iPLA_2_γ-KO mice (grey bars). Reagent concentrations were identical to those described in Figure 1. The number of replicates was at least 6 for both WT and iPLA_2_γ-KO mice. * *p* < 0.05; *** *p* < 0.005; # *p* < 0.001 compared to J_TBHP_. ns, No significant differences between the rates were found in mitochondria isolated from iPLA_2_γ-KO mice.

The results presented so far are consistent with hydroperoxide-dependent activation of iPLA_2_γ, leading to the release of free FAs, which then interact with ANT. This causes an increase in the proton conductance of the inner mitochondrial membrane [40], monitored as an increase in the rate of respiration (Figure 2) [34,36]. Next, we tested whether this mechanism mediates an antioxidant function of iPLA_2_γ in the brain. Figure 7 shows traces of fluorescence of mitochondria-targeted, superoxide-selective probe MitoSOX following the respiration of isolated brain mitochondria. The addition of TBHP led to a decrease in the rate of MitoSOX fluorescence compared to the baseline (no TBHP), and this effect of TBHP was completely reversed in the presence of a selective iPLA_2_γ inhibitor r-BEL or by ANT inhibitor CATR (Figure 7A). No such qualitative changes were observed in brain mitochondria isolated from iPLA_2_γ-KO mice (Figure 7B).

#### 3.3.2. Extramitochondrial H_2_O_2_ Release Decreases following Redox Activation of iPLA_2_γ

**Figure 8:**
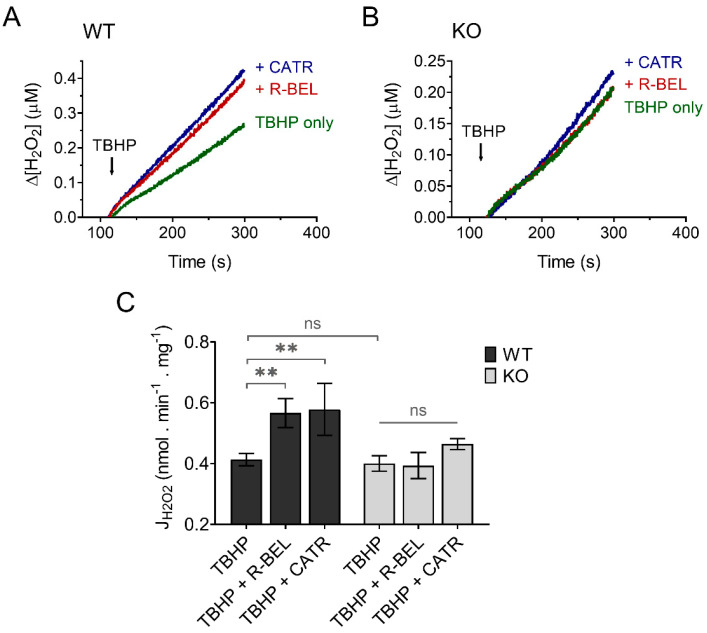
TBHP-dependent activation of iPLA_2_γ leads to decreased H_2_O_2_ formation. Representative traces of extramitochondrial H_2_O_2_ release determined from calibrated resorufin fluorescence in isolated brain mitochondria respiring in the presence of 5 mM of glutamate, 1 mM of malate, 5 mM of succinate (GMS), and 2 μM of auranofin. (**A**) The rate of H_2_O_2_ release in the presence of TBHP (green trace) was inhibited by r-BEL (red trace) and CATR (blue trace). (**B**) The rate of H_2_O_2_ release in the presence of TBHP (green trace), r-BEL (red trace), and CATR (blue trace) in mitochondria isolated from iPLA_2_γ-KO mice. (**C**) The effects of tested compounds on the rates of H_2_O_2_ release of brain mitochondria isolated from WT (black bars) or iPLA_2_γ-KO mice (grey bars). The reagent concentrations were identical to those described in Figure 1. The jump in fluorescence due to the interaction of TBHP with AmplexRed was omitted for clarity. The number of replicates was at least 4 for both WT and iPLA_2_γ-KO. ** *p* < 0.01. ns, No significant differences were found between the rates in mitochondria isolated from iPLA_2_γ-KO mice.

Since H_2_O_2_ readily penetrates mitochondrial membranes, it is routinely assayed quantitatively utilizing the extramitochondrial florescent probe Amplex Ultra Red in the presence of horseradish peroxidase [50]. To minimize the effect of matrix consumers of H_2_O_2_ and thus maximize the amount of H_2_O_2_ released out of mitochondria, we performed our experiments in the presence of the thioredoxin-reductase inhibitor auranofin, which was shown previously to increase the apparent H_2_O_2_ efflux rates [58]. Indeed, under these experimental conditions utilizing both Complex I- and Complex II-linked substrate GMS, the H_2_O_2_ release from mitochondria was relatively fast (Figure 8). The addition of TBHP to brain mitochondria isolated from WT mice resulted in a steady increase in H_2_O_2_ release, which was accelerated in the presence of r-BEL, a selective iPLA_2_γ inhibitor (Figure 8A), which is consistent with the redox-dependent activation of iPLA_2_γ and its simultaneous antioxidant activity. The presence of CATR caused an acceleration of H_2_O_2_ release comparable to that seen in the presence of r-BEL (Figure 8A,C). Again, no such qualitative differences were observed in brain mitochondria isolated from iPLA_2_γ-KO mice (Figure 8B,C).

### 3.4. Antioxidant Role of iPLA_2_γ In Vivo

#### 3.4.1. iPLA_2_γ—Dependent Antioxidant Function Leads to Decreased Protein Carbonyl Content in the Brain

**Figure 9:**
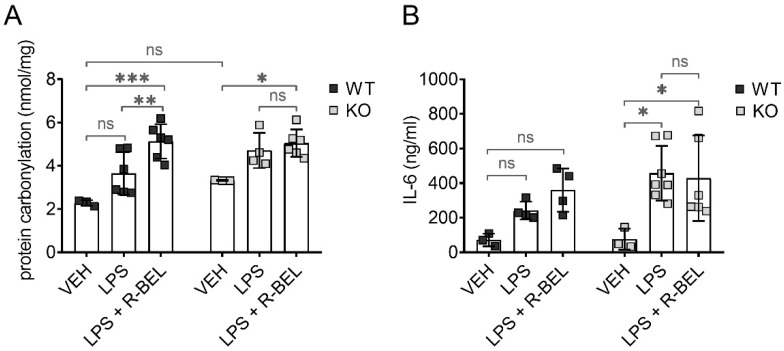
Antioxidant role of iPLA_2_γ in the cytosol and in vivo. Differences in protein carbonyl levels in brain homogenates (**A**) and interleukin-6 levels in serum (**B**) from WT mice and iPLA_2_γ-KO mice. Where indicated, mice were injected with LPS (5 mg/kg body weight) or iPLA_2_γ inhibitor r-BEL (1 mg/kg body weight) and LPS in parallel. * *p* < 0.05; ** *p* < 0.01; *** *p* < 0.005. ns, No significant differences were found between the rates in mitochondria isolated from iPLA_2_γ-KO mice.

To test whether iPLA_2_γ may also play an antioxidant role in vivo, we utilized our previously developed protocols [59] and administered lipopolysaccharides (LPS) to WT and iPLA_2_γ-KO mice together with iPLA_2_γ inhibitor r-BEL. LPS is widely used to study the acute inflammatory response together with acute oxidative stress and causes a dose-dependent increase in protein carbonyl content, which is the most widely used marker of oxidative modification of proteins and a footprint of oxidative stress [60]. Figure 9A shows the levels of protein carbonyls in brain homogenates from WT and iPLA_2_γ-KO mice. The addition of LPS did not lead to statistically significant changes in the protein carbonyl levels in brain homogenates from WT mice, indicating a protective role of iPLA_2_γ. In contrast, LPS treatment caused about a 1.5-fold increase in protein carbonyl levels in iPLA_2_γ-KO mice. The simultaneous administration of LPS and the selective iPLA_2_γ inhibitor r-BEL increased the protein carbonyl content of WT mice to a level similar to KO mice, whereas no further effect of r-BEL administration was observed in homogenates of KO mice. These results support the hypothesis that iPLA_2_γ plays a critical role in the regulation of cellular redox homeostasis and oxidative stress in brain tissues.

#### 3.4.2. iPLA_2_γ–ANT Antioxidant Synergy Decreases the Levels of Inflammatory Marker IL-6

Following LPS injection, the concentrations of IL-6 increased significantly and were higher in iPLA_2_γ-KO mice compared to age-matched WT controls. Moreover, r-BEL, the selective inhibitor of iPLA_2_γ, further elevated IL-6 concentrations in WT mice to the IL-6 levels comparable to those obtained with iPLA_2_γ-KO mice, while the inhibitor had no further effect in iPLA_2_γ-KO mice (Figure 9B). These data demonstrate that the inhibition or ablation of iPLA_2_γ leads to an increase in IL-6 serum levels and are consistent with a proposed antioxidant role of iPLA_2_γ in vivo.

## 4. Discussion

In this work, we demonstrate several consequences of catalytic activities of redox-activated mitochondrial phospholipase iPLA2γ in the brain. We show that (1) FAs liberated by mitochondrial phospholipase iPLA2γ interact with the ANT and play an antioxidant role, (2) iPLA2γ participates in cardiolipin post-biosynthetic remodeling (3) iPLA2γ releases a range of very long-chain PUFAs which may serve as precursors of unique mitochondria-derived lipid mediators of cellular redox signaling.

The molecular mechanism explaining the antioxidant role of iPLA2γ is supported by the ability of ANT to mediate FA-dependent H^+^ transport across the inner mitochondrial membrane, which leads to a mild increase in respiration, corresponding dissipation of the mitochondrial protonmotive force, and consequent attenuation of mitochondrial superoxide formation by the mitochondrial electron transfer system. The decrease in mitochondrial superoxide formation is followed by a decrease in the formation of H_2_O_2_ and consequent oxidants. The antioxidant mechanism based on the iPLA_2_γ-ANT synergy may have consequences extending beyond the respective mitochondrial compartments. This is because mitochondrially-produced oxidants, namely H_2_O_2_ and membrane-permeable electrophilic lipids, can diffuse to cytosolic subcellular compartments and increase the electrophilic tone in the cytosol. This increase has to be compensated by the cytosolic antioxidant mechanisms, reestablishing the redox steady-state. Altogether, the ability of mitochondria to regulate the production of superoxide by the dissipation of the protonmotive force substantiates an antioxidant effect of mitochondria within the intracellular environment [17,45,46].

The conclusions of this study are in contrast to previous data on the putative roles of Ca^2+^-independent phospholipases A2 on mitochondrial ROS production in rat brain mitochondria. It has been concluded that iPLA_2_ does not have a role in the modulation of ROS production in the brain [61]. However, that study probed the effect of externally added DHA (C24:6 PUFA), as the presumed primary product of iPLA_2_ activity, in isolated respiring mitochondria with the attempt to mimic the role of iPLA_2_-liberated fatty acids on the mitochondrial ROS generation. The addition of DHA induced a CATR-sensitive increase in oxygen consumption, which is consistent with ANT-mediated DHA-dependent uncoupling and compatible with our data. However, DHA diminished mitochondrial H_2_O_2_ release only in the presence of succinate, a complex II-linked substrate, but stimulated ROS production in the presence of complex I-linked substrates, which verifies the ability of externally supplied DHA to interact with complex I of the mitochondrial electron transfer system directly, leading to effects associated with lipotoxicity [10]. The results of our study are based on acute activation of iPLA_2_γ, which leads to a local release of fatty acids and thus may avoid the lipotoxicity associated with externally added DHA. In addition, our data show that the profile of iPLA_2_γ-liberated fatty acids is broad and is not limited to DHA. Our conclusions are further supported by the lack of the iPLA_2_γ-dependent antioxidant mechanism in parallel control experiments using iPLA_2_γ-KO mice.

Consistent with previous studies [61], our data show that iPLA_2_γ is intrinsically active in routinely isolated brain mitochondria without the need for added oxidants. We also demonstrate that the presence of NAC during mitochondrial isolation restores the hydroperoxide-induced activation of brain iPLA_2_γ, which may reflect a plausible in vivo situation when the thiol-dependent redox status of the respective mitochondrial compartments controls the activity of iPLA_2_γ. This idea is supported by our previous finding showing that thiol-reducing agents prevent TBHP-induced activation of recombinant reconstituted iPLA_2_γ [35] and further supported by the data obtained in the Oximouse database (https://oximouse.hms.harvard.edu/, accessed on 20 December 2021). The comprehensive and quantitative mapping of the mouse cysteine redox proteome in vivo provided by the database reveals that Cys553 and Cys708 of iPLA_2_γ are oxidized in several tissues, including the brain, suggesting a reversible modification of the respective cysteine residues by hydroperoxides or other electrophiles compatible with oxidizing accessible thiol groups. Coincidentally, a recent study showed that incubation of isolated brain mitochondria with NAC restores the redox state of the glutathione system and increases the levels of S-glutathionylated proteins [62]. Altogether, the results support our hypothesis that iPLA_2_γ contains cysteine residues accessible to reversible oxidative posttranslational modifications, and oxidation of these residues leads to reversible activation of iPLA_2_γ.

**Figure 10:**
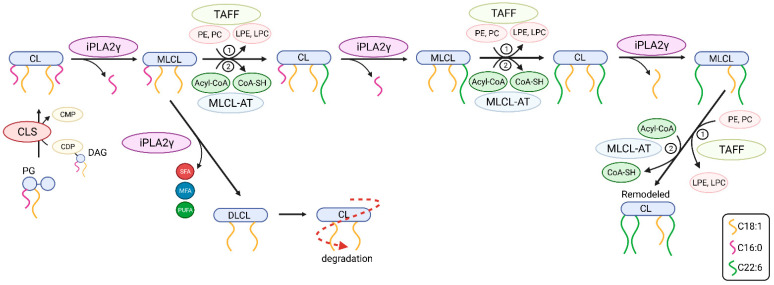
The putative roles of iPLA_2_γ in post-biosynthetic cardiolipin remodeling and degradation. Shown are steps from newly synthesized nascent cardiolipin (CL) to mature CL. The iPLA_2_γ-catalyzed deacylation of CL yields fatty acid (depicted as squiggle) and monolysoCL (MLCL) and is followed by reacylation catalyzed by either (1) Tafazzin transacylase (TAFF) or by (2) MLCL acyltransferase (MLCL-AT). Abbreviations: PG, phosphatidylglycerol; CLS, CL synthase; DAG, diacylglycerol; CDP, cytidine diphosphate; CMP, cytidine monophosphate; SFA, saturated fatty acid; MFA, monoenoic fatty acid; PUFA, polyunsaturated fatty acid; PE, phosphatidylethanolamine; PC, phosphatidylcholine; DLCL, dilysoCL.

Moreover, our data are consistent with the key role of iPLA_2_γ in brain mitochondrial lipid biogenesis. Specifically, the obtained lipidomic data suggest the participation of iPLA_2_γ in cardiolipin post-biosynthetic remodeling (Figure 10). The CL acyl chain composition is tissue-specific, and unlike other tissues, the CL profile in the mammalian brain is temporally dependent, highly diversified, and contains a complex repertoire of CL molecular species [63,64]. iPLA_2_γ is considered, together with iPLA_2_β, a major mitochondrial enzyme involved in cardiolipin remodeling [63]. In addition, iPLA_2_γ has been shown to play a significant role in the generation of MLCL by hydrolysis of short-chain, relatively saturated FAs in the Tafazzin-deficient brain and is involved in the hydrolysis of oxidized CL following traumatic brain injury [33]. Our data show a substantial decrease of five high molecular weight CL species (CL78:14, CL80:12, CL80:14, CL82:16, and CL84:19), and, in contrast, up to a 10-fold accumulation of low molecular weight cardiolipins, namely CL68:1, CL68:2, CL68:4, CL70:6, and CL72:5. These data indicate that iPLA_2_γ aids deacylation of lower molecular weight cardiolipins with predominantly saturated side chains. Our data support an indispensable role of iPLA_2_γ in the remodeling of immature CL to mature species containing high molecular weight and highly unsaturated acyl chains, typical for the brain (Figure 10).

In addition, our detailed lipidomic analyses accurately illustrate the recognized substrate specificity and product pattern of iPLA_2_γ reaction complying with the documented specificity for the PNPLA type of phospholipases [28,65]. At first, we established that in isolated brain mitochondria, iPLA_2_γ liberates both saturated and (poly)unsaturated fatty acids from glycerophospholipids, which is consistent with iPLA_2_γ hydrolyzing both the *sn*-1 and *sn*-2 positions of the glycerol backbone. Second, in the particular case of cardiolipin cleavage, the activation of iPLA_2_γ led primarily to the accumulation of MLCL species, which is consistent with the role of iPLA_2_γ in cardiolipin remodeling. However, we also detected a moderate increase in DLCL, which indicates that iPLA_2_γ can hydrolyze up to two aliphatic chains from cardiolipins. This result is consistent with the finding of Kiebish et al. [66], who detected DLCL in the hearts of mice that were created by crossing strain null for iPLA_2_γ with inducible Taffazin knockdown mice. Those results indicate that iPLA_2_γ plays a critical role in the initial and rapid production of DLCL for cardiolipin remodeling in an acyl-chain-specific manner [66]. From the available lipid and CDP-DG sources within the brain, we conclude that iPLA_2_γ is essential in the synthesis of the high-molecular-weight cardiolipins containing PUFAs in all four acyl chains, which is further documented by our lipidomic analysis of iPLA_2_γ-KO mice.

Our data also demonstrate the ability of iPLA_2_γ to release a range of very long-chain PUFAs, including C24:6, C22:6, C20:5, and C20:4. These PUFAs are metabolically active, participate in the regulation of peripheral immune function, and have been shown to regulate microglia activation in the brain [67]. Specifically, arachidonic acid (C20:4, AA), the main n-6 PUFA, and docosahexaenoic acid, the main n-3 PUFA, are suggested to regulate the molecular signaling of microglia, especially in the context of neuroinflammation and behavior [67]. In addition, both AA and DHA are substrates of cyclooxygenases and lipoxygenases in the brain, resulting in an array of lipid signaling mediators, including fatty acid-derived electrophilic species. These oxidizing compounds are generally considered for their pro-inflammatory properties, but they also have an impact on numerous redox-dependent cell signaling pathways [68]. Previous studies suggested a role for iPLA_2_β in the release of DHA in the brain [69,70,71]. However, the ability of iPLA_2_γ to mediate the release of AA and DHA in the brain has not been fully explored, and suggests an intriguing role for iPLA2γ in mitochondria-produced lipid-dependent intracellular signaling in the brain.

Finally, we show that mice with ablated iPLA_2_γ were more sensitive to stimulation by LPS, as reflected by the concomitant increase in protein carbonyls in brain tissue homogenates and pro-inflammatory IL-6 release detected in the serum. These data support the link between the prevention of pro-oxidative and pro-inflammatory consequences of these treatments and the antioxidant and anti-inflammatory role of iPLA_2_γ in vivo. Then, the proposed iPLA_2_γ-dependent antioxidant mechanism supported by our study may be part of a broad spectrum of physiological functions which iPLA_2_γ plays in the brain, including a causative effect of decreased/compromised activity of iPLA_2_γ leading to neurodegenerative disorders.

## 5. Conclusions

Our data support an antioxidant mechanism in the brain utilizing the release of fatty acids catalyzed by the redox-activated mitochondrial phospholipase iPLA_2_γ. The interaction of iPLA_2_γ-released fatty acids with adenine nucleotide translocase increases proton conductance of the inner mitochondrial membrane and consequently decreases mitochondrial protonmotive force and mitochondrial ROS formation. Our data further indicate a feedback loop in which pro-oxidant milieu or increased oxidative stress activates mitochondrial iPLA_2_γ and initiates iPLA_2_γ-dependent, ANT-mediated antioxidant protection. Moreover, we found an indispensable role of iPLA_2_γ in the remodeling of lower molecular weight immature cardiolipins with predominantly saturated acyl chains to high molecular weight mature cardiolipins with highly unsaturated PUFA acyl chains, typical for the brain.

## Data Availability

Data will be available from the corresponding author upon a reasonable request. The data are not publicly available due to there are still numerous raw data that were collected during the experiments and are not included in this manuscript, because they were considered superfluous. These could be available upon request.

## Supplementary Materials

The following are available online at https://www.mdpi.com/article/10.3390/antiox11020198/s1, Figure S1: Creation of iPLA_2_γ/PNPLA8 knockout mice and the knockout verification. Figure S2: Respiratory control analysis of brain mitochondria isolated from WT and iPLA_2_γ-KO mice. Figure S3: Linoleic acid-dependent increase in respiration in brain mitochondria isolated from WT and iPLA_2_γ-KO mice. Figure S4: Lipidomic analysis of isolated WT and iPLA_2_γ-KO mouse brain mitochondria. Figure S5: Metabolites responsible for sample clustering.
