# Supplementary material for: Antioxidant Role and Cardiolipin Remodeling by Redox-Activated Mitochondrial Ca2+-Independent Phospholipase A2γ in the Brain
Source: Antioxidants (Basel). 2022 Jan 20;11(2):198. doi: 10.3390/antiox11020198 (PMC8868467; doi:10.3390/antiox11020198)
Supplement: Supplementary file 1 [file antioxidants-11-00198-s001.zip › antioxidants-1552722-supplementary.pdf]

antioxidants

Supplementary Figures:

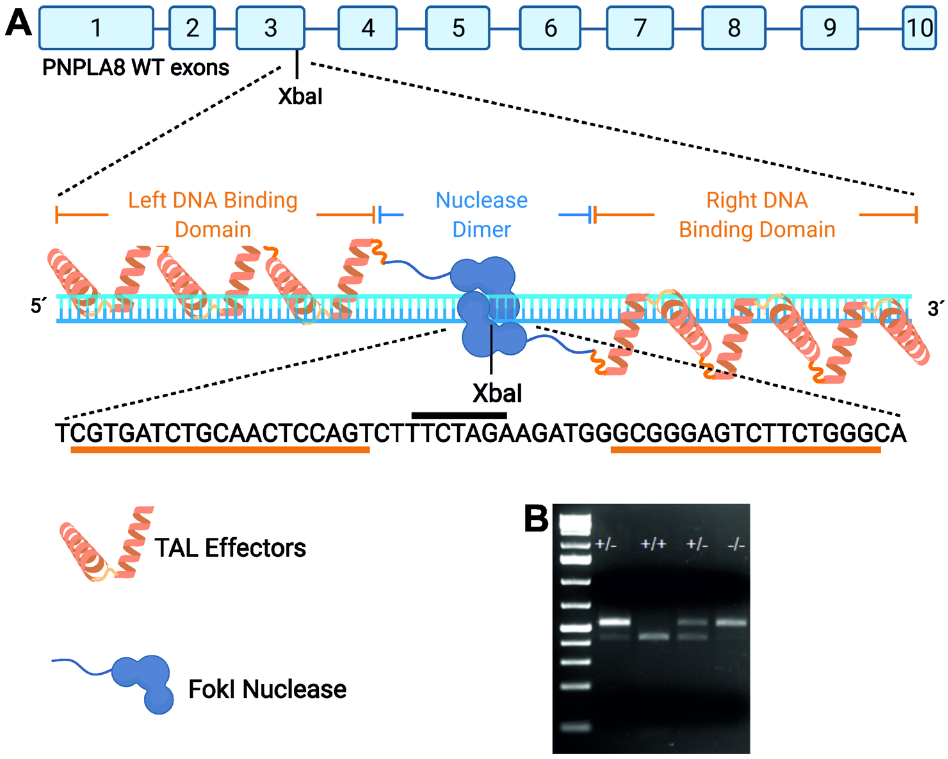

Figure S1: Creation of PNPLA8 knock-out mice and the knock-out verification. (A) Schematic representation of knockout mice construction by targeting the PNPLA8 gene, exon 3 by transcription activator-like effector nuclease (TALEN) FokI. The nuclease cleavage leads to deletion of 13 base pairs, including XbaI restriction site and creation of premature stop codon in exon 4. The scheme was created with BioRender.com. (B) Verification of the PNPLA8 knock-out by PCR restriction-fragment-length polymorphism of the genomic DNA purified from mouse tails; +/+ wild type, +/- heterozygote, -/- knock-out.

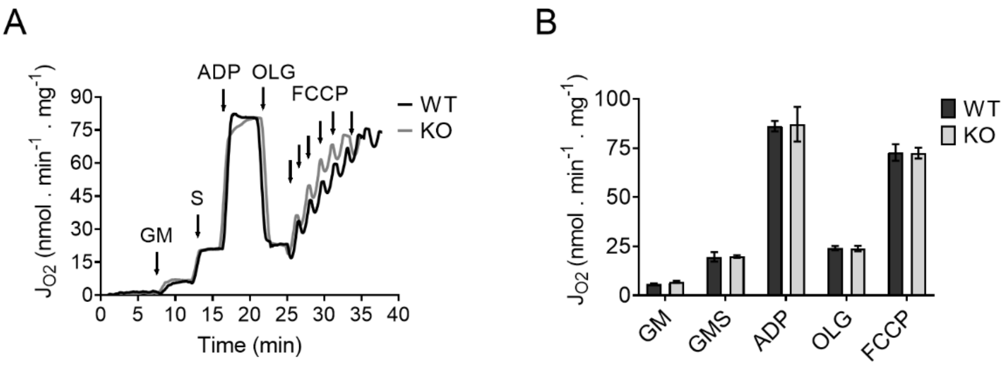

Figure S2. Respiratory control analysis of brain mitochondria isolated from iPLA2γ-WT and iPLA2γ-KO mice. (A) Representative traces of respiration rates (JO2) evolving in time. (B) The effects of various reagents on the rates of respiration of brain mitochondria isolated from iPLA2γ-WT (black bars) or iPLA2γ-KO mice (grey bars) monitored by

changes (Δ) in O2 flux (JO2). Reagents and concentrations: GM (glutamate 5 mM, malate 1 mM), S (succinate 5 mM), ADP (1 mM), oligiomycin (OLG, 1 μM), carbonyl cyanide-4 (trifluoromethoxy) phenylhydrazone (FCCP, 10 nM aliquots).

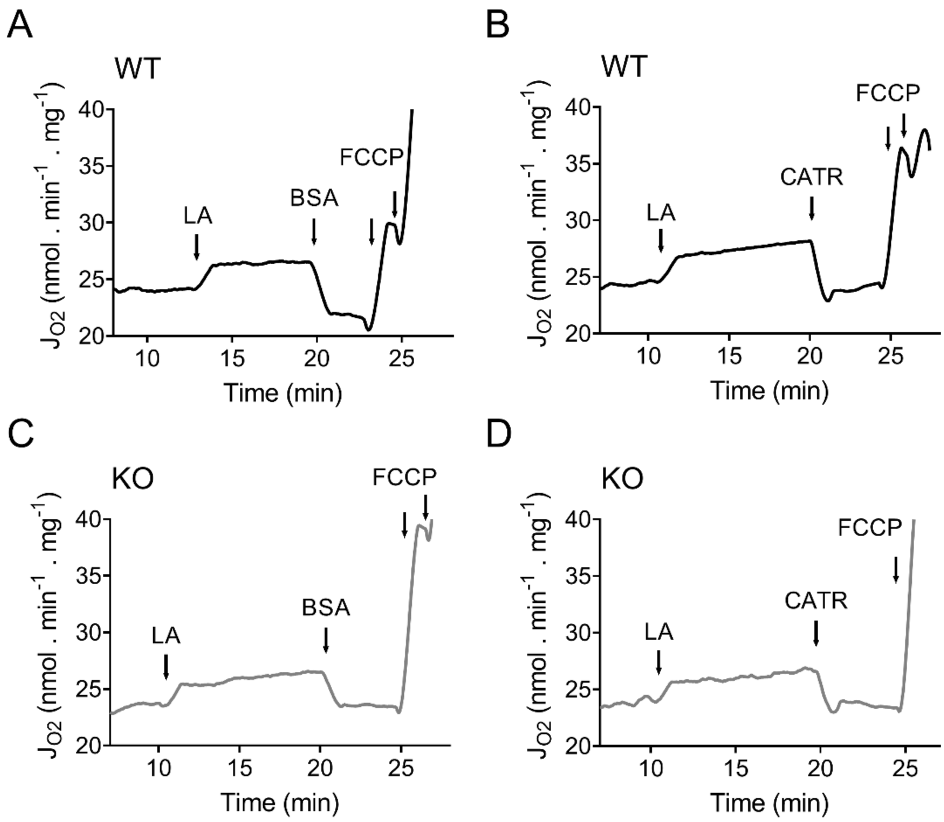

Figure S3. Linoleic acid-dependent increase in respiration in brain mitochondria isolated from WT and iPLA2γ-KO mice. Representative traces are shown for state-4 respiration rates (JO2) evolving in time in the presence of 5 mM glutamate, 1 mM malate, and 5 mM succinate. The increase in respiration caused by the addition of linoleic acid (LA) was reversed by BSA (A) and CATR (B) in brain mitochondria isolated from WT mice, and identically by BSA and CATR brain mitochondria isolated from iPLA2γ-KO mice.

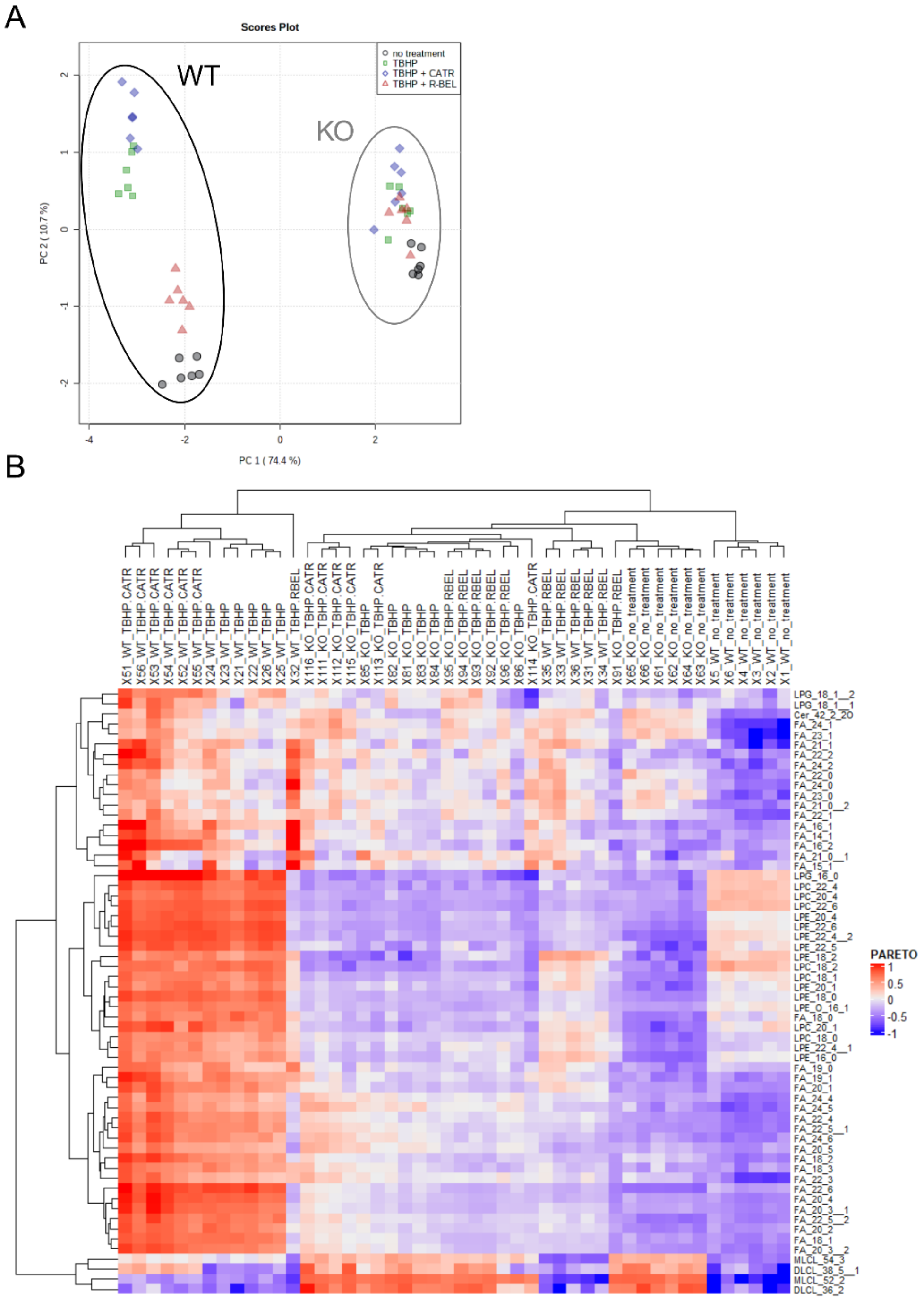

Figure S4. Lipidomics analysis of isolated WT and iPLA2γ-KO mouse brain mitochondria. Principal component analysis scores plot (PCA) of TIC-normalized log_{10}-transformed intensities for WT and iPLA2γ-KO (A). Lipids with loadings values in PC1 > 0.05 in WT and iPLA2γ-KO mouse brain mitochondria (B). Heatmap based on hierarchical clustering presented in dendrograms according to the Euclidean distance. Data are log_{10}-transformed and scaled by Pareto. Isomers are marked with the number after double underscores. Lipidomics data were collected from N = 6 replicates.

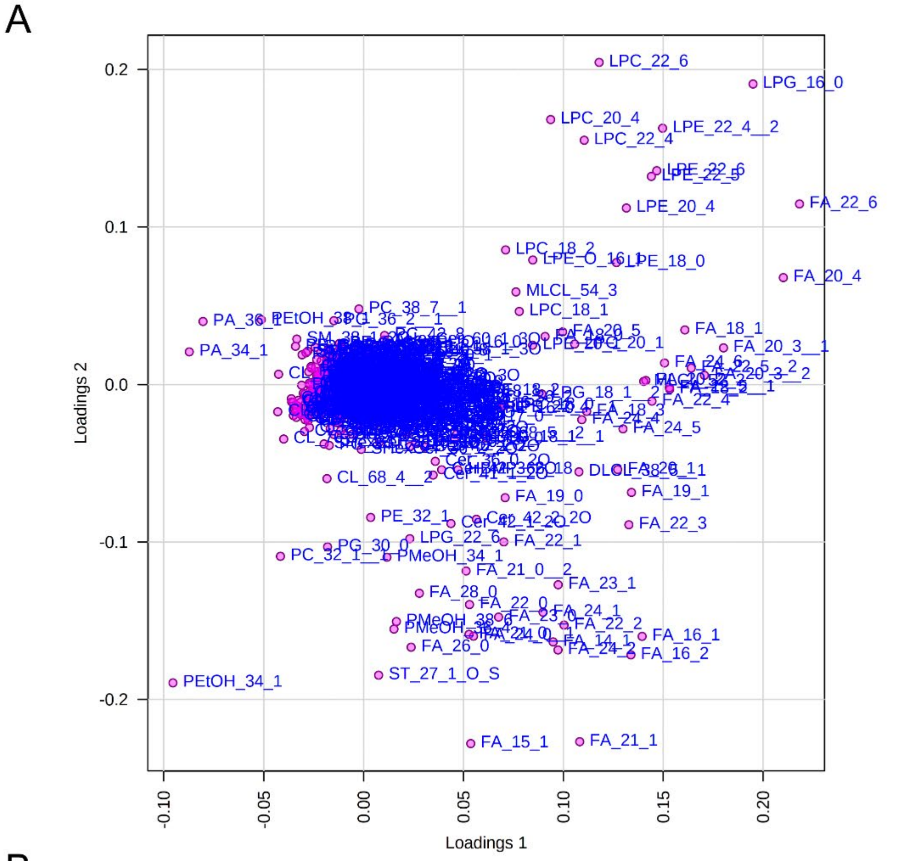

B

Metabolite	PC1	Metabolite	PC1	Metabolite	PC1
FA 22:6	0.21825	LPE 20:4	0.13159	LPE O:16:1	0.08472
FA 20:4	0.21016	FA 24:5	0.12989	LPE 20:1	0.084625
LPG 16:0	0.19499	FA 20:1	0.12723	LPC 18:1	0.077973
FA 20:3 (1)	0.1802	LPE 18:0	0.12662	MLCL 54:3	0.076305
FA 20:3 (2)	0.17066	LPC 22:6	0.11794	LPC 18:2	0.07113
FA 22:5 (2)	0.16403	FA 18:3	0.1117	FA 19:0	0.070887
FA 18:1	0.16086	LPC 22:4	0.11048	LPC 18:0	0.070526
FA 18:2	0.15329	FA 24:4	0.10926	FA 22:1	0.070227
FA 22:5 (1)	0.15309	FA 21:1	0.10822	LPE 22:4 (1)	0.068734
FA 24:6	0.15068	DLCL 38:5 (1)	0.10783	FA 23:0	0.067603
LPE 22:4 (2)	0.14971	LPC 20:1	0.1057	LPG 18:1 (1)	0.061503
LPE 22:6	0.1468	FA 22:2	0.10039	LPE 16:0	0.061096
FA 22:4	0.1444	FA 20:5	0.099675	Cer 42:2:2O	0.056477
LPE 22:5	0.14408	FA 23:1	0.097436	FA 24:0	0.055053
FA 20:2	0.14134	FA 24:2	0.09736	DLCL 36:2	0.054851
MLCL 52:2	0.14026	FA 14:1	0.094923	LPE 18:2	0.053882
FA 16:1	0.13947	LPC 20:4	0.093682	FA 15:1	0.053676
FA 19:1	0.13413	FA 18:0	0.09089	FA 22:0	0.053051
FA 16:2	0.13386	FA 24:1	0.08987	FA 21:0 (1)	0.052809
FA 22:3	0.13283	LPG 18:1 (2)	0.089214	FA 21:0 (2)	0.051343

Figure S5. Metabolites responsible for sample clustering. PCA loadings plot corresponding to the scores plot in Fig. 3A of brain mitochondria isolated from WT mouse (A). List of metabolites with loadings values in PC1 > 0.05 ordered by descending PC1 (B). Isomers are marked with the number in parentheses.
